# Effects of the Physical Recycling of Acrylonitrile–Butadiene–Styrene (ABS) Plastics on the Properties of the Final Product

**DOI:** 10.3390/polym18141716

**Published:** 2026-07-13

**Authors:** Juliana Aristéia de Lima, Ruud Cuypers, Anders Höije, Ignacy Jakubowicz, Richard Sott, Nazdaneh Yarahmadi

**Affiliations:** 1 Department of Polymer, Fiber and Composite, RISE Research Institutes of Sweden, 431 53 Mölndal, Sweden; juliana.aristeia.de.lima@ri.se (J.A.d.L.); richard.sott@ri.se (R.S.); 2 TNO, Kesslerpark 1, 2288 GS Rijswijk, The Netherlands; ruud.cuypers@tno.nl; 3 Division Built Environment, RISE Research Institutes of Sweden, 400 22 Göteborg, Sweden; anders.hoije@ri.se

**Keywords:** acrylonitrile–butadiene–styrene, polybutadiene rubber, physical recycling, dissolution process, material characterization, analysis of impurities

## Abstract

Acrylonitrile butadiene styrene (ABS) is widely used as an engineering plastic, but its extensive use generates a significant amount of waste that is difficult to recycle due to the material’s complex composition. In this study, the physical recycling of ABS using the dissolution technique has been employed to separate the pure copolymer of styrene and acrylonitrile (SAN) from polybutadiene rubber (PBR) and other substances. The relationships between the properties and composition of the original ABS materials were investigated as a starting point and for reference values to evaluate the effects of recycling on the quality and safety of recycled materials. Three different ABS materials were used in the recycling process from which pure SAN polymers were produced. The recycled SANs were then melt-blended with fresh masterbatch. The final ABS materials had the same composition, which facilitated investigation of whether the use of SAN recycled from different sources results in any differences in the properties of the final ABS material. The results showed that all the properties of ABS materials made with recycled SAN are similar regardless of the source of SAN. Several chemical substances were quantified in the original ABS materials and in SAN polymers obtained through the recycling process. The substances were largely removed from all materials except one. The main conclusions from this study are that the quality of ABS materials made with recycled SAN is at the same level as that of virgin ABS and is independent of the source from which SAN comes. This study has also shown that chemical safety is satisfactory because the physical recycling process is able to remove most of the substances that were present in the original ABS materials.

## 1. Introduction

Plastics are inexpensive, lightweight, and durable materials that have substantial benefits in comparison to other materials. They can be readily molded into a variety of products that find use in a wide range of applications. Consequently, the global production of plastics has increased markedly, reaching 430.9 Mt in 2024, of which 54.6 Mt was produced in Europe [1]. However, such high levels of plastic production and usage have generated environmental problems that must be addressed [2]. One of the most important actions currently available to manage these problems is plastic recycling. Increased recycling lowers the demand for virgin plastics which, in turn, results in reduced greenhouse gas emissions from plastic production and lowers the environmental impact of plastic production, use, and waste. However, despite well-functioning recycling systems having the potential to drastically reduce environmental problems, less than 10% of plastic is recycled today globally [1].

There are currently three main approaches for recycling plastics, viz. mechanical, chemical, and physical recycling. However, because of the wide range of recycling and recovery activities in this field, the associated terminology can be inconsistent and confusing. For the sake of clarity, physical recycling in this publication is defined as a “process in which a plastic is subjected to a series of purification steps to separate the target polymer/polymers from other polymers, additives and other added materials such as fibers, fillers, colorants, and contaminants, resulting in recovered polymer(s), which remain largely unaffected by the process and can be reformulated into plastics” [3]. This relatively rarely used recycling method has been used in the current investigation for the recycling of ABS (acrylonitrile–butadiene–styrene) plastics.

ABS is a terpolymer consisting of acrylonitrile, butadiene, and styrene monomers. The polymer is produced by either a mass or emulsion polymerization process. In the first of these processes, styrene and acrylonitrile (AN) react in the presence of a polybutadiene substrate. In the second, ABS is produced in two steps: butadiene is first produced in an aqueous emulsion using radical initiators and emulsifiers, followed by a grafting step in which styrene and acrylonitrile are emulsion-polymerized onto the polybutadiene substrate [4]. The morphological structure of ABS consists of a continuous phase of copolymers of styrene and acrylonitrile (SAN) and a dispersed phase containing mostly polybutadiene rubber (PBR). PBR particles are grafted with SAN to achieve an interaction with the SAN matrix, which endows ABS with high-impact toughness. Consequently, the rubber phase is crucial for the mechanical properties of the final material. Particularly, parameters such as content, particle size, grafting, molecular weight, degree of crosslinking, structure, and properties of the rubbery phase have a great effect on the toughness and other properties [5]. In addition, the final ABS product normally contains one or more of the following additives: antioxidants, colorants, flame retardants, heat stabilizers, impact modifiers, lubricants, nucleating agents, and UV stabilizers. The final product can be produced using various manufacturing processes such as injection molding, which is the most common process; extrusion; blow molding; thermoforming; and 3D printing.

ABS was chosen for this investigation due to its wide and rapidly increasing use. The global market for ABS is expected to reach EUR 42.6 billion by 2027, growing at a CAGR of 6.92% from 2020 [6]. ABS is used as engineering plastic in many applications such as automobile parts, electronic components, medical devices, toys, and consumer goods owing to its high-impact strength, lightweight nature, favorable electric properties, and chemical resistance. Another reason for its selection was that its extensive use generates a significant amount of waste, which is difficult to recycle due to the material’s complex composition. Mechanical recycling is the dominant form of recycling of ABS; in this approach, the waste is ground down, melted, and re-extruded into pellets, which are reformulated and used in new plastic products. However, the main limitation of mechanical recycling is that the recycled materials often have lower performance compared to virgin materials. The main reason for this is degradation caused by thermo-oxidation, thermo-mechanical stress, and polymer chain scission, which mainly affects the rubber part. The presence of contaminants can also be of great importance. According to some reports, even 1% impurities can significantly impair the mechanical properties of the material [7]. In addition, mechanically recycled ABS contains various additives from the original products, which give rise to safety concerns in some applications. For example, the ABS waste from electronic and electrical equipment contains harmful additives such as brominated flame retardants (5–25 wt.%) and heavy metals (0.5–3 wt.%) [8].

The drawbacks of mechanical recycling can be avoided by using a physical recycling process based on dissolving the polymer waste in a suitable solvent; filtering out any insoluble contaminants, additives, and other unwanted substances; and recovering the pure SAN polymer through re-precipitation and drying [9,10]. By optimizing dissolution process conditions such as choice of solvent, polymer concentration, dissolution temperature, and time, the structure and molecular weight of the SAN polymer can be maintained while minimizing the environmental impact. This process of recycling ABS waste contributes to the development of a circular economy, reduces the environmental impact of waste and promotes sustainable development. The ongoing development of eco-friendly solvents has great potential to further reduce the environmental impact and enhance the scalability of this method.

The main purpose of this study was to evaluate the effects of the physical recycling process on the quality, safety, and functionality of ABS materials made with recycled SAN from various waste sources and a masterbatch (MB) formulated for a final application. The quality of recycled ABS depends heavily on its composition, purity, and morphology. To evaluate the effect of a recycling process, it is important to have detailed knowledge of the relationships between the composition and properties of virgin ABS, which has also been investigated here as a starting point. This knowledge is needed to be able to make a fair comparison between virgin and recycled materials. The investigation therefore consists of two parts: The first part includes an evaluation of the properties of industrially manufactured ABS materials based on two completely different SAN polymers with varying PBR content. The second part includes the investigation of starting ABS materials manufactured with small-scale equipment followed by the physical recycling process and manufacturing of new ABS materials from recycled SAN and MB using the same manufacturing equipment.

## 2. Materials

### 2.1. Materials Included in This Investigation

The materials included in this study can be divided into two groups: virgin ABS materials with different rubber content and the materials that were used in the recycling experiments. In the first group, two series of ABS materials with varying rubber content based on two different SAN polymers were manufactured by Trinseo (Hoek-Terneuzen, The Netherlands) using industrial manufacturing equipment for the investigation of how material composition affects properties. The SAN polymers are designated SAN 1 with a low AN content and high molecular weight (M_w_) and SAN 2 with a high AN content and low M_w_. The materials were used in the development of analytical methods for the determination of rubber content and in the tests to determine the dependence of properties on rubber content and type of SAN.

**Figure 1:**
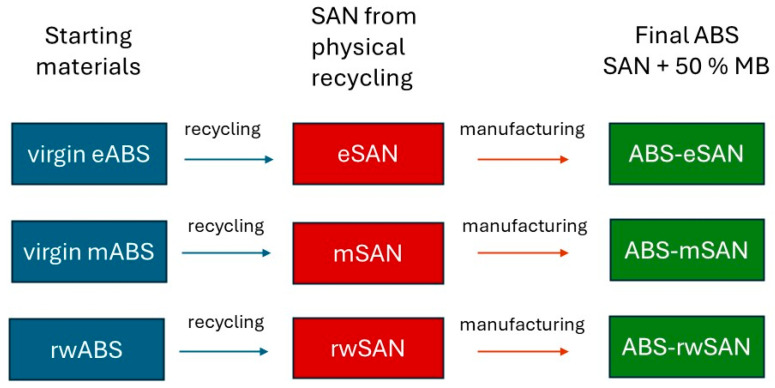
The second group of investigated materials and material flows.

The second group of materials which is included in the recycling study can be divided into three categories: starting ABS materials, SANs obtained from the starting ABS materials using the physical recycling process based on TNO Möbius dissolution technology, and the final ABS materials produced from the recycled SANs and one masterbatch (MB) by melt processing using a micro-compounder (Xplore Instruments, Sittard, The Netherlands) (see Figure 1). Three different starting materials were used in the recycling experiments viz. virgin eABS, virgin mABS, and mechanically recycled ELV waste ABS (rwABS).

The second group of materials used in the recycling experiments are provided in Figure 1.

In all final ABS materials in this category, both the PBR content (25 wt.%) and the content of additives were at the same level, which created similar conditions for the comparison of mechanical and other properties between different ABS samples. This approach provided an opportunity to compare the effect of SAN recycled from different sources on the properties of the final product.

### 2.2. Physical Recycling

In the recycling experiments, TNO Möbius dissolution technology was used. This technology is based on a selective dissolution process in which the polymer is separated from impurities, additives, and contaminant polymers [11]. It is an innovative solvent-based technology developed to address the challenges of plastic recycling with the objective of recovering and decontaminating polymers that can be re-compounded to plastic. TNO’s Möbius process utilizes a selective solvent-based approach for the dissolution of specific polymers from pretreated waste plastics streams (sorted and washed).

**Figure 2:**
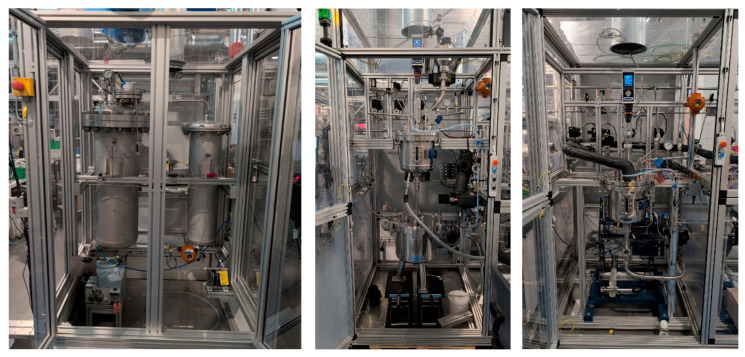
TNO Möbius Leto Solvent Supply Module (SSM, **left**), Dissolution Module (DSM, **middle**) with filter unit (as can be seen mounted below the main vessel), and AntiSolvent Module (ASM, **right**) as used.

To enable flexibility and thus choose the best configuration for use cases and projects, a modular approach was used. The process was therefore divided into larger unit operations and modules created from these. These modules hold all the directly needed equipment and operating system (software) to support the modules’ smooth operation. The different modules that together created the recycling process were as follows: the solvent supply module (SSM), the dissolution module (DSM) combined with the filtration unit, the antisolvent module (ASM), and the solvent recovery module (SRM) (see Figure 2).

The purpose of the solvent supply module was to safely store and supply the right amount of solvent at the right pressure and temperature for introduction into the DSM. This was accomplished by using a supply tank equipped with a positive displacement pump. The supply tank can be refilled with solvent at will and is connected to the DSM for solvent introduction into the dissolution process at the desired pressure and temperature.

The purpose of the combined dissolution and filter module was to dissolve ABS from ABS-rich waste and separate SAN from its additives, especially PBR. The waste was added as shredded material of the proper size and quantity into a closed, heated, and pressurized vessel, and solvent from the SSM was flowed in in a controlled manner. After dissolution of the soluble part of the waste (SAN), the solution was filtered through a coarse filter removing all larger non-dissolved particles. Afterwards, the solution entered the dedicated filter unit to remove various sizes of particles from the polymer solution by bed filtration. After dissolution at an elevated temperature, the vessel was cooled. The speed of filtration was adjusted by adjusting the pressure difference between the lines before and after the filter unit. Ideally, a clear solution exited the filter unit flowing to the antisolvent module (ASM).

The purpose of the ASM was to introduce the polymer solution in a controlled manner into an amount of antisolvent to initiate precipitation of the dissolved polymer. For the present case, methanol was used as an antisolvent. The solidified polymer (SAN) was then extracted from the setup after draining the residual solvent and opening the vessel.

A solvent recovery module (SRM) was used as the last step. The purpose of the SRM was to recover solvent, using a condensing heat exchanger. The residual collected solvent was heated under a vacuum and subsequently cooled. Cooling for the heat exchanger was provided by a cooling bath. The condensed solvent was collected in a vessel for further processing and reuse.

The ABS waste that entered in the DSM was dissolved under elevated temperatures and pressures by the solvent etylacetat (EtOAc) which was introduced from the SSM. The solvent slowly dissolved the SAN over time (several hours) at a temperature of 80–120 °C under stirring. The solvent then passed through a coarse and fine filter using positive pressure to remove undissolved particles (in the filter unit). The solution then passed to the ASM unit where SAN was precipitated with methanol.

The solid, precipitated, recycled SAN was obtained by emptying the vessel and removing the material before the drying step. The dry, solid material (in powder or chunks) was further used in this study for analyses and production of the final ABS materials.

An important part of the development of the Möbius recycling process was solution recovery as it affects both the economics and environmental impact of the process. The solvent recovery and reuse were centered around the Solvent Recovery Module (SRM) of the TNO Möbius Leto setup. This module is specifically designed to enable circular use of the process solvent, which in the present case was primarily ethyl acetate (EtOAc). The SRM operates by collecting residual solvent streams from upstream units (notably after precipitation and separation) and subjecting them to a thermal-vacuum treatment. In this step, the solvent is heated under reduced pressure, promoting evaporation at lower temperatures, which is beneficial for energy efficiency and for avoiding thermal degradation of solvent components. The evaporated solvent is then routed to a condensing heat exchanger, where it is cooled using a dedicated cooling bath (from the utilities module), allowing it to condense back into liquid form. The recovered liquid solvent is subsequently collected in a storage vessel, making it available for further processing and direct reuse in the dissolution process.

In terms of reuse, the process is designed as a closed-loop solvent system, where recovered EtOAc can be reintroduced into the SSM for subsequent dissolution cycles. This approach minimizes fresh solvent demand and reduces environmental emissions. Although the SRM is not permanently integrated in the current configuration of the TNO Möbius Leto setup, its function was already conceptually embedded in the process design and is fully functioning. Overall, the combination of vacuum-assisted evaporation, condensation, and storage enables efficient recovery while maintaining solvent quality suitable for repeated use in selective dissolution. The SRM for industrial operation is still under development but in a future scaled-up version, the recovery efficiency target is 95–98%.

The TNO Möbius dissolution process used in this study is not yet fully developed for industrial use. There are several parameters that still need to be optimized for the process to be acceptable as an industrial process. EtOAc was chosen as the solvent, but two important parameters must still be optimized which are the maximum concentration of ABS and the dissolution temperature within the range 80–120 °C. The dissolution time which is a function of ABS concentration and temperature is an important parameter for the process to be industrially feasible. Time and temperature affect both the costs related to the recycling process and the risk for thermally induced degradation. As a result of the recycling process, a SAN polymer is obtained which is precipitated using methanol as an antisolvent. The last parameter that also needs to be optimized is an industrially efficient filter system.

However, the work of optimizing the recycling process for industrial use does not affect the results presented in this publication.

### 2.3. Manufacturing of Materials

The melt processing of ABS materials significantly influences the final product’s properties. Process parameters such as temperature, shear rate, and cooling rate determine the polymer’s viscosity, molecular integrity, internal stress, shrinkage, etc. Each parameter plays an important role in the formation of microstructure and stress distribution, thus affecting the product’s mechanical properties [12]. In this study, only small amounts of recycled SAN were available for investigation, which is why all ABS materials based on recycled SAN were produced with a micro-compounder. Consequently, all the material properties of ABS made with recycled SAN were evaluated for materials produced with a micro-compounder while the properties of the first group of ABS materials were evaluated for materials produced with industrial equipment. The absolute values of the mechanical properties presented here may therefore differ between the materials obtained with laboratory and industrial equipment. However, the most important objectives of the investigation were to show how the properties vary with the rubber content and type of SAN and to compare the quality of the ABS materials made with recycled SAN from different waste sources. In this context, the results should be seen primarily as relative rather than absolute.

#### 2.3.1. Industrial-Scale Manufacturing of ABS Materials by Trinseo

Trinseo, a company that offers a broad line of plastics and binders in many areas of application, provided the virgin raw ABS materials and molded test specimens for this investigation. It also produced the masterbatch (MB) and several eABS compounds with various proportions of PBR, as well as the virgin eABS and mABS materials that were used as input to the TNO Möbius process.

#### 2.3.2. Small-Scale Manufacturing by RISE

Before processing, ABS was dried at 80 °C for 4 h in an oven. The samples studied regarding the quality of the recycled ABS (see Figure 1) were prepared by mixing virgin or recycled SAN with the fresh masterbatch (MB) containing PBR grafted onto SAN and standard additives. The materials were then processed in a micro-compounder (Xplore MC 15 HT), which is a small-scale twin-screw extruder, at 220 °C, 50 rpm. The materials were maintained in the machine for 1 min using a recirculation channel, and then transferred to an injection molding attachment (Xplore IM 12) and molded to test bars of the shape specified in ISO 527-2 type 5A, producing rectangular specimens with dimensions of 80 × 10 × 4 mm.

## 3. Methods

The quality of ABS materials is determined by a combination of mechanical, thermal, physical, and chemical properties which are largely shaped by the ratio of the three monomers. ABS is a so-called rubber-toughened material in which a brittle SAN polymer is blended with rubber particles to create a tough material. Consequently, the morphology of ABS materials together with the volume fraction of the rubbery phase and rubber particle size and orientation are very important factors controlling the mechanical properties of ABS [13]. The following sections describe the methods used in this study to evaluate the key properties that determine the quality of ABS materials.

### 3.1. Morphology

The morphology of ABS is represented by a continuous SAN phase in which grafted PBR particles are dispersed. Morphological analysis was performed using scanning electron microscopy (SEM) on test specimens from bars used for impact testing. These bars were fractured in liquid nitrogen and then immersed in an aqueous solution of 2 wt.% osmium tetroxide for 48 h. Then, the specimens were washed, dried, and coated with gold to make the samples electrically conductive during SEM imaging, using a Zeiss Supra 40 VP instrument. Backscattered electron (BSD) images of the materials were obtained using an accelerating voltage between 10 and 20 kV. To view the contrast differences clearly between the phases and to measure the particle size, the original SEM images were converted to negative images using ImageJ^®^ software.

SEM statistics of particle size were calculated by analyzing individual images to determine distribution using the median diameter, which was obtained by measuring the particle dimensions from an image, including the diameter for roughly spherical particles and length and width for irregular ones. The statistical analysis of hundreds of particles, presented in histograms, provides data on the mean (D) and number-based percentages (using ImageJ^®^ and Origin^®^ software).

### 3.2. Mechanical Testing

Mechanical properties were evaluated using tensile and impact strength measurements. Ten test specimens were used when the first group of ABS materials manufactured with industrial equipment was evaluated. Only 5 test specimens were used when the second group of materials manufactured with a micro-compounder were evaluated.

The impact tests were performed at 23 °C and −30 °C using a Resil Impactor and following the ISO 179-1 standard “Determination of Charpy impact properties”.

Tensile tests were performed at room temperature on a Zwick/Roell Z100 system according to ISO 527-2 and using a 5 kN load cell. The sample size was Type 5A, with a preload of 0.1 MPa and test speed of 10 mm/min. The test speed during the measurements of tensile modulus was 1 mm/min.

### 3.3. Thermal Analysis

Thermo-gravimetrical analysis (TGA) experiments were performed on a Mettler Toledo TGA1 instrument. The test materials were heated from room temperature to 800 °C in a nitrogen atmosphere at a heating rate of 10 K/min and the weight of the sample was registered as a function of the heating temperature.

Determination of Vicat softening temperature (VST) was performed according to ISO 306 method B50 using 50 N of force and a heating rate of 50 K/h. It is measured as the temperature at which the sample is penetrated exactly 1 mm by a flat-ended circular indenter of 1 mm^2^ in cross-section.

### 3.4. Rheology by Melt Mass Flow Rate (MFR) Measurements

The MFR of the materials investigated in this study was measured using the test method ISO 1133-1, procedure A, with the following test conditions: temperature 220 °C and load 10 kg. The MFR was determined as the mass extruded over a specified time under prescribed conditions and calculated as grams per 10 min. The standard recommends using a half-size die if the MFR is >75 g/10 min. This was not applied in this study in order to be able to make a direct comparison between materials with different rheological properties, which increased the measurement uncertainty for the material with a high MFR.

### 3.5. Rubber Content Analysis

Rubber content is one of the most important factors determining the physical, mechanical, and rheological properties of ABS materials. For this reason, many different methods have been developed over the years to determine the PBR content in ABS as reported by Zamani et al. [14]. Furthermore, in this study attempts were made to determine the rubber content but without using chemicals or solvents. Consequently, two different methods were selected for the analysis, namely pyrolysis–gas chromatography/mass spectrometry (Py-GC/MS) and Fourier Transform InfraRed (FTIR) spectroscopy.

**Figure 3:**
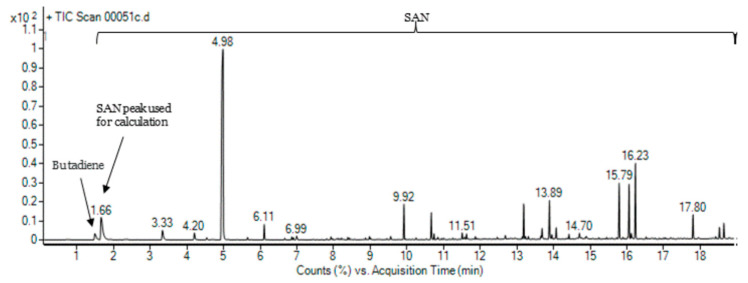
Example of a pyrogram from ABS.

The result of the chemical analyses using Py-GC/MS was a pyrogram which is a characteristic mass spectrum of a material (see Figure 3), with the intensity of the products set against the retention time. Mass spectra were recorded to enable identification of the chemical structure of the materials. The pyrograms show one peak from PBR and several peaks corresponding to SAN. A calibration curve was created by plotting the ratio of the area of the PBR peak and a selected peak at 1.67 min from SAN against the % PBR contents.

**Figure 4:**
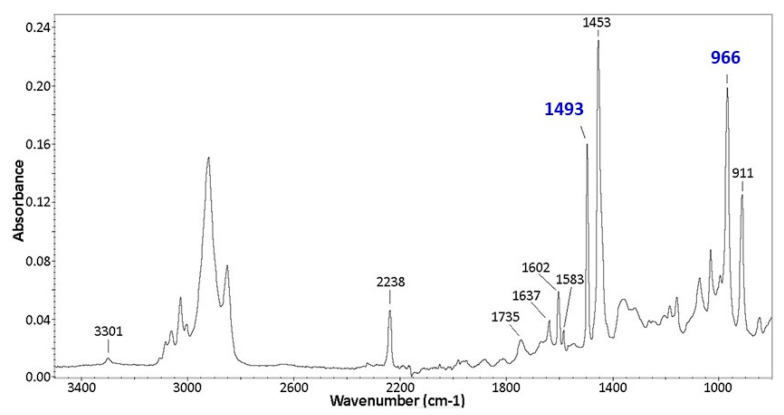
Example of an FTIR spectrum of ABS.

In the second method, FTIR spectra were collected using a Nicolet iS50 FTIR spectrometer equipped with an all-reflective diamond-attenuated total reflection (ATR) attachment (see Figure 4). A total of 32 scans collected between 4000 and 400 cm^−1^ were averaged to reduce noise. The resulting spectra were compared with the literature.

While quantitative analysis by IR spectroscopy has some limitations, performing comparisons of the spectra obtained from materials containing known amounts of PBR makes it possible to construct a calibration curve and thus determine the content of PBR in an unknown material. ABS materials with known rubber contents were analyzed with FTIR. The ratios of the absorbance peaks’ intensities between the band appearing at 966 cm^−1^, corresponding to PBR, and the band at 1493 cm^−1^, corresponding to the aromatic ring vibration in polystyrene, which is frequently used as the internal reference, were plotted against PBR content.

### 3.6. Chemical Analysis of Substances

Chemical analysis was performed with a focus on material compliance according to the European Union’s REACH regulation (EC) No 1907/2006, the Toy Safety Directive 2009/48/EC, and the relevant product requirements for the automotive industry.

A chemical analysis of the samples was performed by GC-MS, XRF, ICP-OES, and IC to detect the remaining substances and SVHCs (Substances of Very High Concern) present before and after the recycling process. Both screening analysis and methods to determine the concentration of individual compounds such as pollutants or additives in ABS were used, in combination with migration tests of certain elements. The analysis methods were chosen in view of product safety requirements in the areas of hygiene and automotive and toy safety with a focus on the detection, identification, and quantification of selected substances in the rABS.

#### 3.6.1. Materials and Reagents

Dichloromethane (>99%, stabilized with 2-metyl-2-buten 20 ppm), methanol (99%), nitric acid (70%), and hydrogen peroxide (30%) were purchased from Merck (Darmstadt, Germany). Styrene (>99%) and Irganox 1076 (>98%) were obtained from TCI Europe N.V. Certified reference standard solutions of DEHP (Bis(2-ethylhexyl) phthalate), triphenyl phosphate, and bisphenol A were purchased from Neochema GmbH.

#### 3.6.2. X-Ray Fluorescence (XRF)

Elements heavier than sodium were screened by handheld XRF, Thermo ED-XRF, and Niton XL3t, focusing on halogen concentration determination. The concentration of bromine was determined using external standards of ABS samples with known concentrations in the range of 20–150 mg/kg.

#### 3.6.3. Inductively Coupled Plasma–Optical Emission Spectrometry (ICP-OES)

The samples were dissolved with a mixture of nitric acid and hydrogen peroxide by microwave digestion. The metal content was determined through inductively coupled plasma–optical emission spectrometry (ICP-OES) using external standards.

#### 3.6.4. Determination of Total Fluorine Content

Quantification of total fluorine was performed using pyrohydrolytic combustion of samples, followed by fraction collection of the fluoride formed and IC detection, i.e., combustion ion chromatography (CIC). The instrument used was an XPREP C-IC from Trace Elements, together with an IC from Metrohm.

#### 3.6.5. Migration Tests

The migration of elements from the samples to water was tested according to the European standard EN 71-3, Safety of toys—Part 3: Migration of certain elements. All the elements except chromium (VI) and organic tin were determined by inductively coupled plasma–optical emission spectrometry (ICP-OES). Organic tin was determined in sample parts made from a material that can contain organic tin compounds, with a total concentration of tin at a level that could exceed the limit for the part analyzed. Chromium (VI) was determined by ion chromatography (IC) using inductively coupled plasma–mass spectrometry (ICP-MS). Chromium (VI) was determined in sample parts with a total concentration that could exceed the limit for the part analyzed.

#### 3.6.6. Characterization of Volatile Organic Compounds by GC-MS (Gas Chromatography–Mass Spectrometry)

The GC-MS analysis was performed on an Agilent GC:MS 6890N equipped with 5975 Inlet XL MSD with Triple-Axis Detector, and a DB-5MS column (30m, 0.250 mm, 0.25 mm), using helium carrier gas at 1.2 mL/min and an inlet temperature of 280 °C. The temperature program started at 35 °C for 3 min followed by heating at 15 °C/min to 320 °C, maintaining the final temperature for 8 min. The mass spectrometer operated in EI mode at 70 eV, in the scan range *m*/*z* 32–650 Da.

The samples were extracted in dichloromethane in a sonic bath for 30 min at 40 °C. The extracts were diluted in methanol and analyzed by GC-MS after removal of the solid polymers by centrifugation. The compounds detected by GC-MS were identified using the NIST database of mass spectra, and the concentrations were estimated in decane equivalents. The external decane standards showed a linear standard concentration range between 1 and 80 mg/L. The method detects compounds with boiling points between 100 °C and approximately 380 °C, and the detected compounds are divided into three categories: oligomers, solvents, and unknowns.

Initially, the screening analysis by GC-MS revealed the presence of Bisphenol A, styrene, triphenyl phosphate, DEHP, and Irganox 1076 in the mechanically recycled ABS samples. These compounds were therefore determined specifically by external standards, using four standard points between 2 and 80 mg/L in the calibration curve.

#### 3.6.7. Characterization of Residual Solvents by Headspace GC-MS

For the detection of compounds with boiling points lower than 100 °C, the samples were heated in sealed headspace vials at 120 °C for 30 min before injecting the headspace on the GC-MS. The compounds detected by GC-MS were identified using the NIST database of mass spectra, and the concentrations were estimated in decane equivalents based on an external standard.

The GC-MS analysis was performed on an Agilent 7890B GC system equipped with 5977B MSD and a DB-5MS column (30 m, 0.250 mm, 1,0 mm), using helium carrier gas at 0.9 mL/min and an inlet temperature of 280 °C. The temperature program started at 35 °C for 2 min followed by heating at 10 °C/min to 280 °C, maintaining the final temperature for 5 min. The mass spectrometer operated in EI mode at 70 eV, with a scan range of *m*/*z* 30–650 Da.

## 4. Results and Discussion

### 4.1. Properties of Virgin ABS Materials

#### 4.1.1. Determination of Rubber Content

To evaluate the effects of recycling on the quality of a recycled material, it is important to have detailed knowledge about the relationships between the properties, morphology, and composition of the original materials. Rubber content is one of the important factors determining the physical, mechanical, and rheological properties of ABS plastics [15]. In addition to rubber content, the size of the rubber particles is also known to be an important parameter especially affecting the fracture toughness of rubber-modified materials [16]. Consequently, the development of a suitable method to determine rubber content in ABS also formed part of this study. Two different analytical methods were selected for the analysis of rubber content.

**Figure 5:**
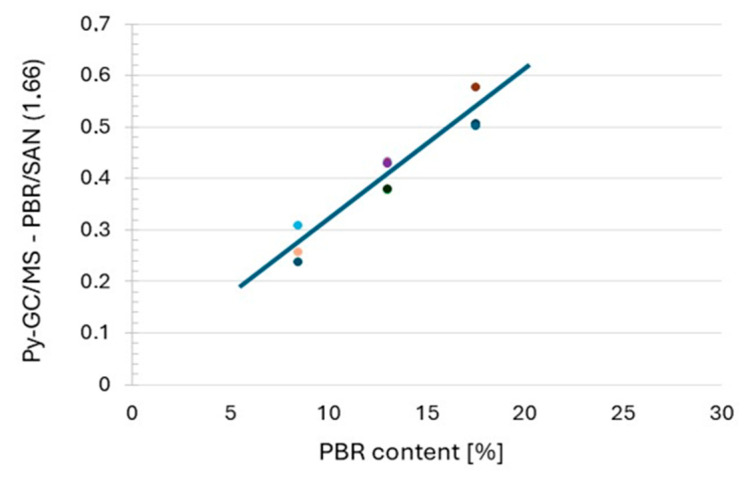
Ratio of area for PBR/SAN peak eluting at 1.67 min against the % contents of PBR.

Calibration curves using Py-GC/MS were successfully created to determine the content of PBR in ABS. However, the area ratio of PBR/SAN depends on the settings and temperatures during pyrolysis. A calibration curve therefore must be created in connection with the analysis of PBR in unknown samples. The Py-GC/MS calibration curve is presented in Figure 5. The measurement uncertainty for the highest rubber contents was about ±1.5% and for the lower contents it was ±1%.

**Figure 6:**
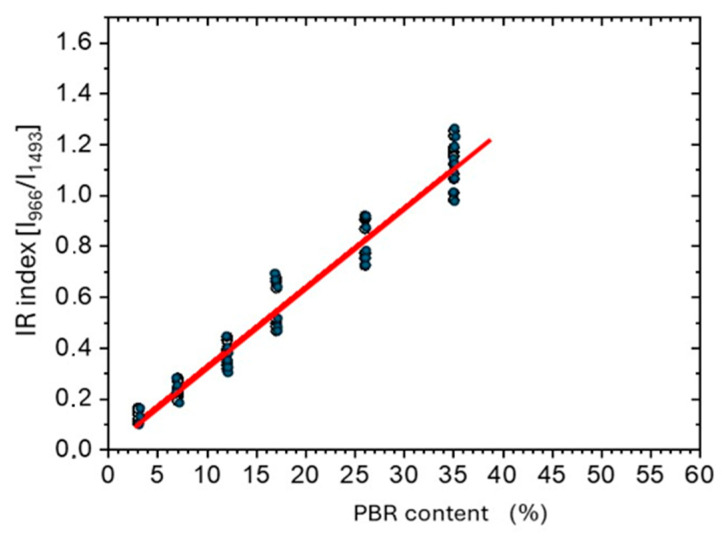
IR index as the ratio between band intensities at 966 cm^−1^ and 1493 cm^−1^ vs. the % contents of PBR.

Additionally, another calibration curve was created using FTIR. While sample preparation and the FTIR analysis itself are relatively simple, the complex structure of ABS containing grafted rubber and the different linkages in PBR, such as 1,4-cis/trans and 1,2-vinyl, which give rise to different IR signals, can complicate direct quantification and contribute to greater measurement uncertainty. The measurement uncertainty for the highest rubber contents (35%) was about ±3% and decreased with decreasing rubber content. The FTIR calibration curve is presented in Figure 6.

#### 4.1.2. Mechanical Properties

**Figure 7:**
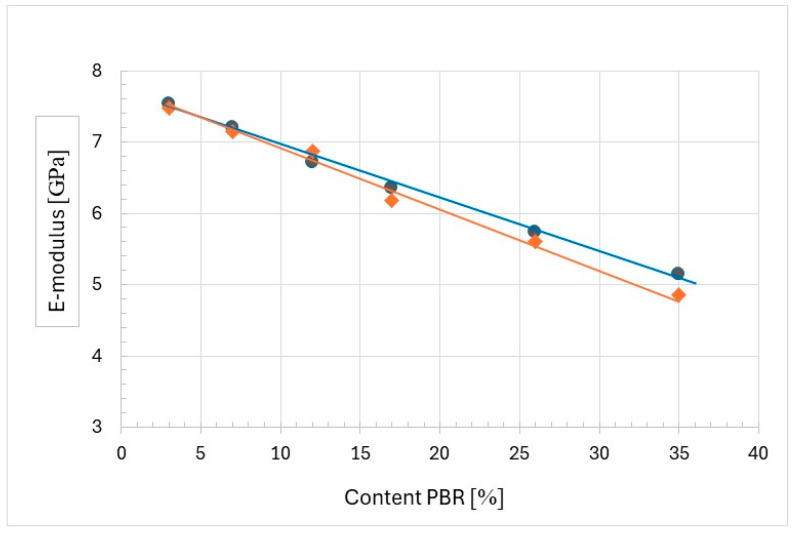
Young’s modulus vs. PBR content for two ABS materials based on SAN 1 (high M_w_ and low AN content) (blue line) and SAN 2 (low M_w_ and high AN content) (orange line).

As mentioned before, mechanical properties depend on the morphology of the ABS materials together with the PBR content and rubber particle size and orientation. However, SAN’s properties can also affect the mechanical and rheological properties of ABS, especially the acrylonitrile (AN) content and molecular weight (M_w_) of the SAN [17]. In this study, two different SAN polymers were used, namely SAN 1 (low AN content, high M_w_) and SAN 2 (high AN content, low M_w_), to produce ABS materials with various proportions of PBR. It is generally recognized that both an increased AN content and a higher M_w_ of SAN can lead to a slightly higher E-modulus. Both SAN polymers used in this study resulted in ABS materials with almost the same E-modulus. This result is in agreement with the theory that the modulus of a blend with dispersed spherical rubber particles depends mainly on the rubber particle volume fraction alone and is the most important parameter controlling the modulus values of ABS resins. The E-modulus of the two ABS materials decreased linearly with increasing rubber content as expected for both materials, as shown in Figure 7.

The different effects of the two SAN polymers used are more visible in properties such as tensile strength and elongation at break as shown in Table 1. Generally, a higher AN content in the SAN phase increases rigidity because of stronger polar interactions and thus a higher tensile strength of the ABS material but often leads to a decrease in the elongation at break. Conversely, increasing the PBR content decreases tensile strength but increases the elongation at break. These effects can also be seen in Table 1, where the ABS based on SAN 2 has a lower elongation at break but a higher tensile strength at a lower PBR content compared to the ABS based on SAN 1.

#### 4.1.3. Rheological Properties

**Figure 8:**
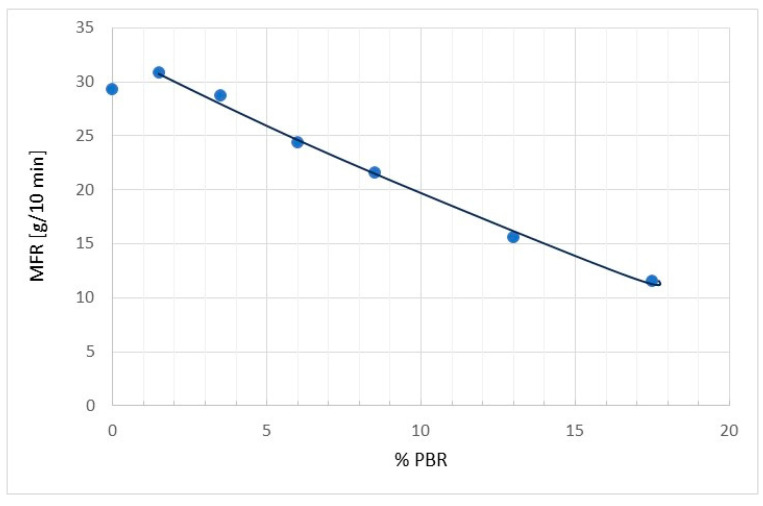
Melt flow rate vs. % PBR for ABS material containing SAN 1.

**Figure 9:**
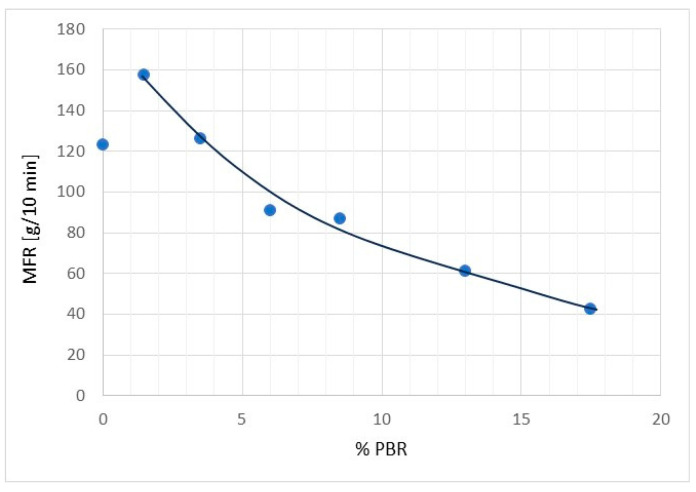
Melt flow rate vs. % PBR for ABS material containing SAN 2.

Optimal processing of plastic materials is important for the properties of the final product. One of the fundamental properties to consider when selecting processing parameters is rheological behavior. Knowledge of this parameter is vital to carry out correct extrusion or injection molding of the polymer. A common method for measuring rheological properties is measuring melt flow rate (MFR). In Figure 8 and Figure 9, the results of MFR as a function of PBR content in the two previously described SAN matrices are presented. It is seen that the MFR of ABS plastics is inversely proportional to its rubber content. As the rubber content increases, the viscosity of the ABS melts increases, resulting in a decrease in MFR, which means that the material is more difficult to process.

It is worth pointing out the large difference in MFR between the ABS based on SAN 1 and SAN 2 is mainly due to the differences in M_w_. The MFR of both SAN polymers increases by adding small amounts of PBR. Thereafter, MFR decreases with increasing PBR content.

#### 4.1.4. Morphological Properties

ABS is a multiphase polymer blend with structural and compositional properties whose effects are complex and interdependent. The mechanical properties of ABS are one of many properties affected by the rubber phase volume fraction, particle size and size distribution, and structure, which in turn are affected by the manufacturing process. Industrial synthesis of ABS is performed mainly via emulsion or mass polymerization routes. Due to differences in these polymerization processes, these two main ABS material types exhibit different morphologies concerning PBR particle content, shape, and size distribution.

**Figure 10:**
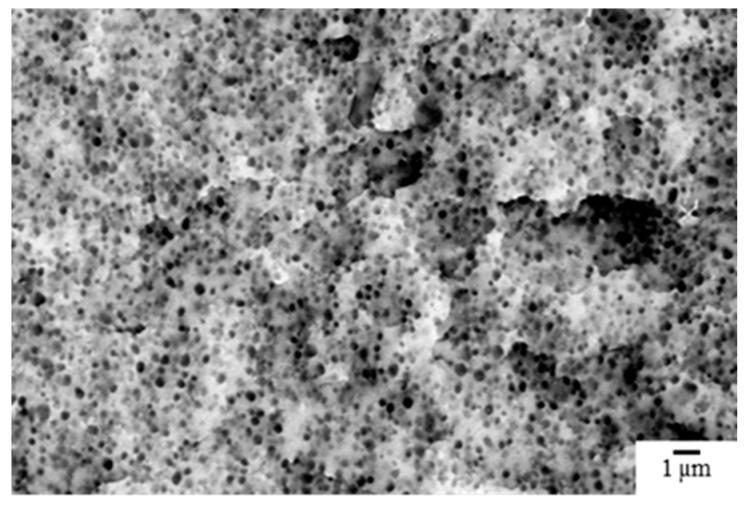
SEM micrograph of eABS indicating the shape and dispersion of rubber particles in the matrix, visible as dark dots.

**Figure 11:**
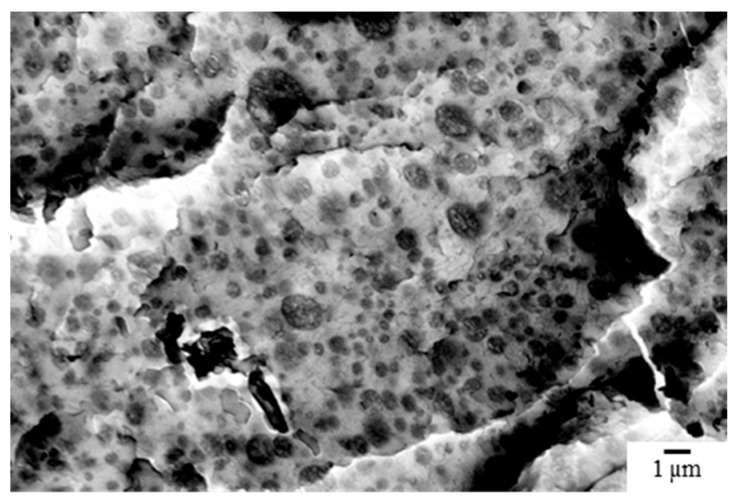
SEM micrograph of mABS indicating the shape and dispersion of rubber particles in the matrix which exhibit a characteristic “salami” structure.

Commercial eABS contains a significantly higher amount of grafted ABS (gABS) compared to mABS. It also includes a compounding step of the first polymerization product with pure SAN; thus, it can be regarded as a blend of gABS with SAN [18]. In the mass process, preformed PBR is dissolved in the styrene and acrylonitrile monomers which polymerize around the rubber phase, thus causing mABS to have a higher amount of SAN inclusions in the PBR particles. This results in a significantly larger spherical diameter while maintaining a low PBR content, resulting in the well-established “salami” morphology of mABS. Examples of SEM images of eABS and mABS are shown in Figure 10 and Figure 11.

#### 4.1.5. Thermal Properties

**Figure 12:**
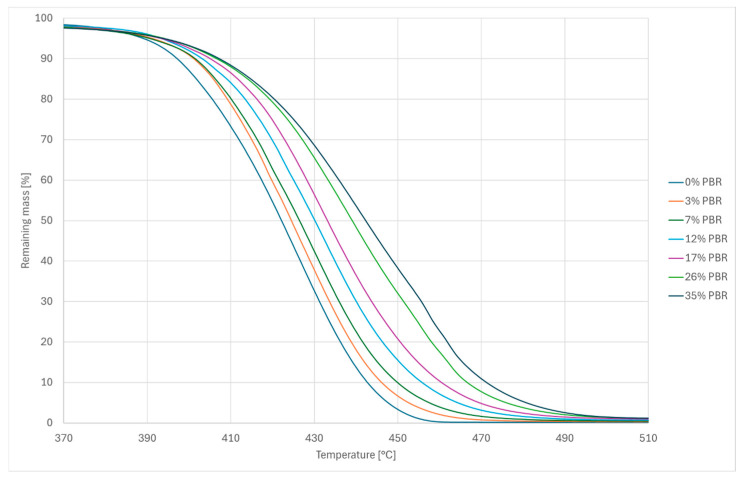
TGA analyses of ABS containing SAN 2 plus various amounts of PBR.

Thermal analysis provides a thermal fingerprint for materials. In this investigation, TGA was used to study the thermal decomposition of ABS materials containing various amounts of PBR. The TGA curves are presented in Figure 12. All TGA curves show a one-stage weight loss between 390 °C and 500 °C. The analyses clearly show that the decomposition temperature increases with increasing PBR content in the material. This relationship is in good agreement with the results of Hitachi, who reported that the decomposition of PBR is reflected in the high temperature range while the decomposition of styrene is reflected in the low temperature range [19].

Another important thermal property is Vicat softening temperature (VST), which serves as an indicator of when the material begins to lose its structural rigidity under a specified load. ABS materials based on SAN 1 (low AN, high M_w_) and SAN 2 (high AN content, low M_w_) with various proportions of PBR were also used in this test. As shown in Table 2, the composition of SAN does not seem to affect VST significantly while it decreases with increased PBR content as expected. In theory, VST should increase with an increased M_w_ and also increase with increased AN content. Since a SAN polymer with a low AN and high M_w_ and a SAN polymer with a high AN and low M_w_ were used, the effects of these two parameters seemed to cancel each other out, resulting in the same effect on the VST.

### 4.2. Effect of Physical Recycling on Properties of Final ABS Materials

#### 4.2.1. The Effect on Mechanical Properties

The mechanical properties of ABS materials are highly dependent on several technical factors such as material composition, manufacturing method, and processing temperature. To find out whether the mechanical properties of the final ABS are affected by the source from which the SAN is recycled, three different ABS materials were manufactured with the same composition and the same manufacturing method. The only difference is that the SAN was obtained via a TNO Möbius recycling process from three different ABS materials, namely eABS, mABS, and rwABS, the latter of which is a mechanically recycled ABS from ELV (end-of-life vehicle) waste. The tensile properties of the original and recycled materials are reported in Table 3, while the impact strength is reported in Table 4.

Mechanical tests show that ABS materials made with recycled SAN give similar results regardless of the source of the SAN. It is also clear that the mechanically recycled ABS has poorer impact strength; however, after removing PBR and adding new additives, the material acquires the same properties as the others.

#### 4.2.2. The Effect on Viscosity

As demonstrated in Figure 7 and Figure 8, MFR is strongly dependent on the rubber content and the molecular weight of SAN. It is also affected by the size and hardness of the rubber particles. In the ABS materials based on recycled SANs, the rubber particles are the same, which results in materials with almost the same viscosity, as shown in Table 5.

#### 4.2.3. The Effect on Morphology

**Figure 13:**
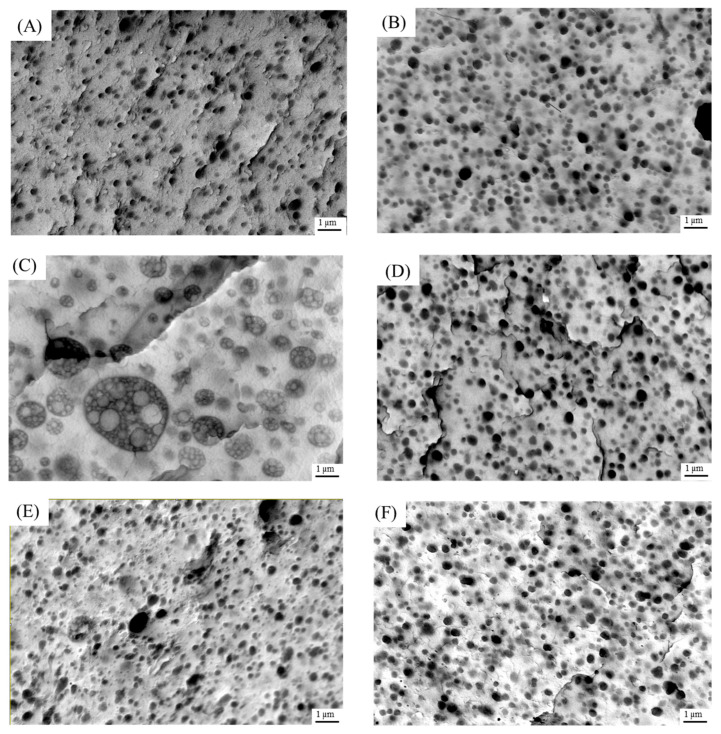
SEM micrographs of (**A**) virgin eABS, (**B**) eSAN + 50% MB, (**C**) virgin mABS, (**D**) mSAN + 50% MB, (**E**) rwABS, and (**F**) rwSAN +50% MB. Magnification: 20,000×.

It is known that the size and shape of rubber particles are important parameters that affect, in particular, the mechanical properties of ABS materials. PBR particles’ shape, distribution in the matrix, and size distribution were studied by SEM. The SEM micrographs are shown in Figure 13, where the images on the left side are of the original ABS materials, while those on the right side are of the ABS materials based on recycled SAN. Micrographs of the original ABS materials show different morphologies: Firstly, there is a very distinct difference between eABS and mABS, where mABS exhibits a characteristic “salami” structure. Secondly, there is a difference in particle size distribution. Of course, the images from the ABSs with recycled SAN show the same morphology because the PBR particles from original materials are removed while the new PBR particles are from the same MB used in manufacturing.

**Figure 14:**
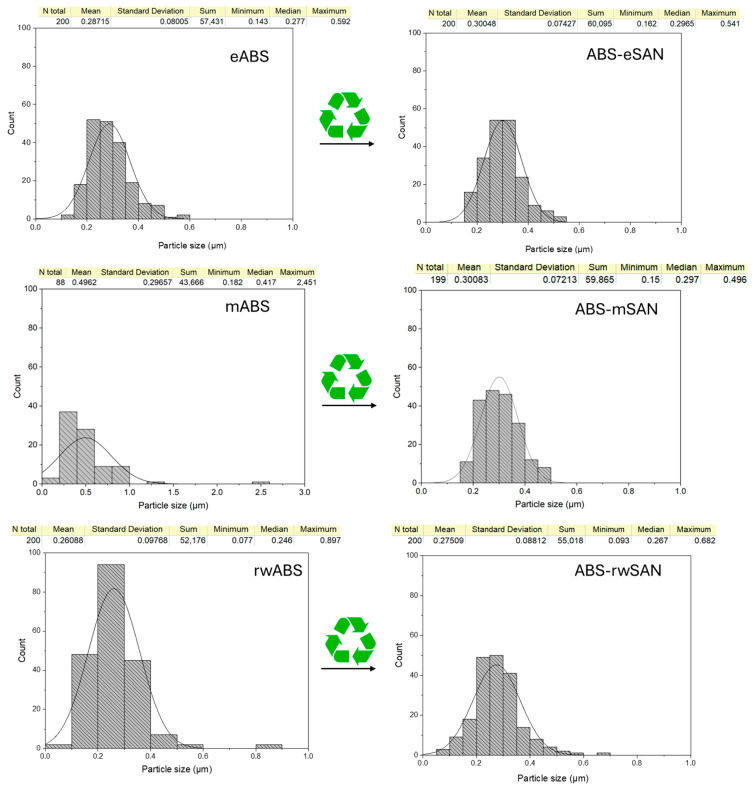
Particle size distribution before and after recycling.

It can be seen in Figure 14 that the particle size distributions in eABS and the ABS with recycled eSAN are almost the same, ranging between 0.1 and 0.6 microns. Naturally, the two other ABS materials with recycled SAN also have similar particle size distributions because all SANs were blended with the same MB. Of course, mABS differs due to its “cell-type” particles containing internal SAN inclusions showing broader and larger particle size distribution ranging between 0 and 1.3 microns.

#### 4.2.4. Analysis of Impurities and Other Substances

Post-consumer plastic waste, even if it has been well sorted and cleaned beforehand, can contain contaminations and unwanted substances. It is generally accepted that physical recycling has great potential to remove contaminants. However, the optimal removal of contaminants during the dissolution-based recycling of ABS depends on several factors, including solvent selection, polymer concentration, and process configuration (temperature, filtration method, etc.). Table 6 shows the concentration of certain substances in the original ABS materials and the concentration of the substances in the corresponding SANs after physical recycling.

The compounds detected in the eABS and mABS samples before recycling were styrene, Irganox 1076 and various oligomers (mainly identified as ABS and SAN dimers and trimers), and unknown compounds. The compound concentrations were significantly reduced in the eSAN and mSAN samples after recycling, except for solvent concentration, which increased due to the solvent residues from the recycling process. However, the small amount of solvent should not have any practical significance because it evaporates in the subsequent compounding step without affecting the properties of the material. On the other hand, the reduced content of oligomers in recycled SAN could affect the rheological properties of the polymer melt during processing.

The concentration of styrene was reduced to levels clearly below the LOQ (limit of quantification) for both samples apart from Irganox 1076 in eSAN, which was detected at approximately 8 mg/kg. Bisphenol A, triphenyl phosphate, DEHP, cadmium, and bromine were detected in the rwABS sample at concentrations between 40 and 320 mg/kg. The recycling to the rwSAN sample resulted in a decrease to concentrations between 28 and 130 mg/kg. The styrene, Irganox 1076, and oligomer concentrations also showed a minor decrease. Overall, the recycling to the rwSAN sample was less effective in decreasing the compounds detected, compared to the concentration reduction observed for the recycling of samples eABS and mABS.

## 5. Conclusions

The main purpose of this study was to evaluate the effects of the physical recycling process on the quality, safety, and functionality of recycled ABS materials for final applications. The crucial properties and functionality of an ABS plastic product stem from its polymer composition and whether suitable additives are utilized. To evaluate the effect of a recycling process, it is important to have detailed knowledge of the relationships between the composition, morphology, and properties of virgin ABS materials in order to make a fair comparison. This study therefore consists of two parts: one that focuses on mapping these relationships for virgin ABS and one that evaluates the ABS made with recycled SAN.

The composition of ABS is fundamental to its properties, where variations in the ratios of the monomers acrylonitrile, butadiene, and styrene directly determine its mechanical, thermal, and processing characteristics. Since ABS materials with an unknown composition are often handled in the recycling process, it is important to have simple and quick methods for determining the PBR content. In this study, two different methods were therefore evaluated for the determination of rubber content without using chemicals or solvents, namely pyrolysis–gas chromatography/mass spectrometry (Py-GC/MS) and Fourier Transform InfraRed (FTIR) spectroscopy. Both methods made it possible to construct calibration curves that can be used to determine the rubber content of an unknown ABS material.

The investigation of material properties consists of two parts. In the first part industrially manufactured ABS materials based on two completely different SAN polymers with varying PBR content were used. The relationships between the properties and composition of the original ABS materials were investigated as a starting point and for reference values to evaluate the effects of recycling on the quality and safety of recycled materials.

The second part includes the investigation of starting ABS materials manufactured with small-scale equipment followed by the physical recycling process and the manufacturing of new ABS materials from recycled SAN and MB using the same manufacturing equipment.

Using industrially manufactured materials, it was investigated how important properties vary with rubber content and SAN composition using two series of virgin ABS materials with varying rubber content and based on two different SAN polymers (SAN 1 (low AN content, high M_w_) and SAN 2 (high AN content, low M_w_)). Both series of ABS materials exhibited almost the same tensile E-modulus, which decreased linearly with increasing rubber content. Differences between the ABS based on SAN 1 and SAN 2 could be noticed in properties such as tensile strength and elongation at break, where the ABS based on SAN 2 had a lower elongation at break but a higher tensile strength at lower PBR contents compared to the ABS based on SAN 1. The difference between these two SANs is significantly greater when it comes to rheological properties. The MFR of ABS with SAN 2 (low M_w_) is four times higher than that of the ABS with SAN 1 (high M_w_). The MFR of both ABS materials with various SAN polymers decreases with increasing PBR content. On the other hand, no clear difference between the ABSs with SAN 1 and 2 can be measured regarding VST (Vicat softening temperature) but both show decreasing VSTs with increasing PBR content. Another thermal characteristic that was investigated was the decomposition temperature measured by TGA, which increases with increasing PBR content in the material.

The effects of physical recycling were evaluated using ABS materials manufactured with small-scale laboratory equipment. The physical recycling of ABS using a dissolution technique separates the relatively pure SAN polymer from mainly PBR, additives that were used in the original materials, and possible contaminants. The recycled SAN is then melt-blended with the fresh masterbatch (MB) containing PBR grafted onto SAN and standard additives using a micro-compounder. In this study, eABS, mABS, and mechanically recycled ELV waste ABS were used as starting materials. The final ABS materials based on recycled SANs had the same composition, which created an opportunity to compare the effect of SAN recycled from different sources on the properties of the final ABS materials. This information is important for recycling companies because it means that if there is no difference, the company does not need to separate waste from different sources.

The mechanical properties of ABS materials made with recycled SAN are similar regardless of the source of the SAN. It is also clear that mechanically recycled ABS has poorer impact strength; however, after removing PBR and adding MB, the material acquires the same properties as the others. Since ABS materials based on recycled SANs contain the same PBR and additives, the MFR and morphology are the same. Of course, mABS morphology as a starting material differs because the special “cell-type” particles in mABS are removed during the recycling process and replaced by emulsion-type rubber particles.

An important part of the investigation was to evaluate how effective the recycling process is at removing various substances. This information is crucial to ensure health and regulatory safety as well as purity and mechanical performance. The optimal removal of various substances in a dissolution-based recycling process depends on several factors, both chemical and physical. The most important factors are the choice of solvent, the concentration of the polymer, and the process conditions prevailing during the separation. Several substances were quantified in the original ABS materials and in the SAN polymers obtained from the physical recycling process. Chemical analysis indicates that the TNO Möbius process successfully separates the product from monomers, oligomers, elements, and additives by using a small-scale apparatus. Styrene, which was detected in virgin eABS and mABS, was effectively removed by the process. Bisphenol A was detected in ABS from mechanically recycled ELV waste and was partially removed. Oligomers and antioxidants were largely removed from all materials except the ELV waste. Bromine was only found in the ABS waste from ELV, which was reduced from 270 to 40 mg/kg after the physical recycling process. Migration tests have shown that the amounts of elements and styrene are well below the limits specified in the European toy directive. The reduction in compound and element concentrations by physical recycling is significantly higher for the virgin samples compared to that of mechanically recycled ABS waste, which we believe is due to changes in the parameters in the recycling process.

This study has shown that the quality of ABS materials made with recycled SAN is at the same level as that of virgin ABS and is independent of the source from which it comes. It has also shown that the chemical safety is satisfactory because the physical recycling process is able to remove most of the substances that were present in the original materials. We believe that the information in this article can be critical when making decisions to invest in an industrial process for the physical recycling of ABS waste.

## Figures and Tables

**Table 1:** Tensile properties of virgin ABS with different rubber content.

PBR Content	Tensile Strength [MPa]		Elongation at Break [%]	
[%]	SAN 1	SAN 2	SAN 1	SAN 2
3	44.3	72.3	2.7	1.2
7	45.7	55.6	4.4	2.3
12	46.4	40.9	6.3	2.9
17	42.3	41.1	5.3	2.5
26	39.1	37.5	6.2	4.0
35	36.7	23.0	6.2	4.1

**Table 2:** Vicat softening temperature [°C].

PBR Content [%]	eABS, SAN 1	eABS, SAN 2
0	101.4	100.4
17	96.3	96.2
26	94.6	94.8
35	92.1	93.9

**Table 3:** Tensile properties of the recycled ABS materials.

Sample	Original ABS			ABS with Recycled SAN		
	E (GPa)	σ (MPa)	ε (%)	E (GPa)	σ (MPa)	ε (%)
mABS	1.35	36.4	23.7	1.32	41.8	16.8
eABS	1.48	44.6	12.1	1.41	38.4	12.2
rwABS	1.30	50.0	10.2	1.39	37.7	17.6

**Table 4:** Impact strength [kJ/m^2^].

Sample	Original ABS		ABS with Recycled SAN	
	23 °C	−30 °C	23 °C	−30 °C
mABS	18.5	10.4	20.5	11.7
eABS	19.0	9.0	21.6	12.2
rwABS	10.7	5.2	22.2	14.7

**Table 5:** MFR of the original virgin eABS, mABS, and mechanically recycled rwABS (the middle column) and ABS materials based on SANs recycled from the respective original materials (right column) [g/10 min].

Sample	Original ABS	ABS Based on Recycled SAN
mABS-	10.8	23.8
eABS-	27.0	26.3
rwABS-	27.2	24.1

**Table 6:** The content of certain substances before and after physical recycling indicated in [mg/kg].

Substance [mg/Kg]	eABS	eSAN	mABS	mSAN	rwABS	rwSAN
Styrene	200	<0.5	170	<0.5	120	90
Bisphenol A	<1	<1	<1	<1	320	130
Triphenyl phosphate	<1	<1	<1	<1	42	4.1
DEHP	<5	<5	<5	<5	40	28
Irganox 1076	1400	10	2300	<10	3100	470
Bromine	<20	<20	<20	<20	270	40
Cadmium	<25	<25	<25	<25	43	<25
Solvents and unknowns	530	2600	270	450	310	1800
Oligomers	9500	79	3800	170	3000	2800

## Data Availability

The original contributions presented in this study are included in the article. Further inquiries can be directed to the corresponding authors.

## Abbreviations

The following abbreviations are used in this manuscript:

AN	Acrylonitrile
ABS	Acrylonitrile–Butadiene–Styrene
gABS	grafted ABS
mABS	ABS obtained by mass polymerization
eABS	ABS obtained by emulsion polymerization
vABS	Virgin ABS (original material)
wABS	ABS waste (post-consumer and post-industrial)
rwABS	Mechanically recycled wABS
SAN	Copolymer of styrene and acrylonitrile
mSAN	SAN from mABS after the Möbius process
eSAN	SAN from eABS after the Möbius process
rwSAN	SAN from rwABS after the Möbius process
PBR	Polybutadiene rubber
MB	Masterbatch (50% SAN + 50% PBR)
XRF	X-ray fluorescence
ICP-OES	Inductively coupled plasma–optical emission spectrometry
GC-MS	Gas chromatography–mass spectrometry
IC	Ion chromatography
